# Establishing risk-based recall interval for caries management among 11-12-year-old Pakistani children

**DOI:** 10.1186/s12903-022-02383-z

**Published:** 2022-08-13

**Authors:** Muhammad Taqi, Ishak Abdul Razak, Norintan Ab-Murat, Syed Jaffar Abbas Zaidi

**Affiliations:** 1 Department of Community Dentistry, Dow Dental College, Dow University of Health Sciences, Karachi, 74200 Sindh Pakistan; 2 Faculty of Dentistry, MAHSA University, Bandar Saujana Putra, 42610 Jenjarom, Selangor Malaysia; 3 Department of Community Oral Health & Clinical Prevention, Faculty of Dentistry, Universiti Malaya, Kuala Lumpur, Malaysia; 4 Department of Oral Biology, Dow Dental College, Dow University of Health Sciences, Karachi, 74200 Pakistan

**Keywords:** ICDAS, Caries increment, Recall interval, Cariogram

## Abstract

**Background:**

This study aims to investigate the rate of caries increment among 11-12-year-old Pakistani children over 18 months using modified International Caries Detection and Assessment Systems II (ICDAS) and subsequently establish an appropriate dental recall interval for our targeted population according to their caries risk intensity.

**Methods:**

A prospective longitudinal study was conducted in Bhakkar, Punjab, Pakistan. The 226 children from seven schools of Bhakkar with the highest student enrolment were conveniently selected. Caries risk assessment was performed using a computer-based reduced Cariogram program. Caries increment among cavitated lesions was measured by modified Beck's method or adjusted caries increment. Two ICDAS II cutoffs were created for the analysis of cavitated lesion (ICDAS code 3–6) and cavitated plus non-cavitated lesion (ICDAS code A-6).

**Results:**

At the risk assessment stage, 39.8% of the children were classified as low risk, 30.5% as medium risk, and 29.7% as high risk. Caries increment at both cutoff points increased with caries risk at all follow-ups. The highest caries increment was recorded at the third follow-up among high-risk children at cutoff 3–6 (1.95 ± 3.18) and A-6 (4.01 ± 4.31). However, the lowest caries increment was recorded at the third follow-up among low-risk children at cutoff 3–6 (0.18 ± 1.42) and A-6 (1.11 ± 3.33).

**Conclusion:**

Based on the study findings for Pakistani children with cavitated lesions, the recommended risk-based recall interval for caries management is 18 months for those with low and medium caries risk and six months for those with high caries risk. On the other hand, recommendations for risk-based recall intervals for caries management in non-cavitated and cavitated lesions are six months for low-risk, moderate risk and high-risk for Pakistani 11-12-year-old children.

## Introduction

A dental recall visit is defined as ‘the scheduled appearance of a patient, previously examined in better oral health’ [1]*.* The recall interval is the period between two consecutive routine oral examinations, measured in months or years [1]. A recall examination involves a series of clinical activities performed during this visit, such as detection of early signs and symptoms of oral diseases, primary and secondary prevention, advice on dental compliance, and screening of intraoral indicators of systemic disorders [2–7]. There has been a growing debate on the appropriate timing of the recall interval for a dental checkup, influenced by the increasing demand for oral health care, the management of human health resources, and the availability of evidence about appropriate recall visits. Traditionally, the recall interval between two dental examinations recommended by oral health practitioners for primary care is six months, and it is still practiced in many countries [8, 9]. However, the clinical and cost-effectiveness of six months interval between dental recalls have been challenged [8, 9]. The practice of fixed dental recalls can lead to misuse of scarce resources and supplier-induced demand [10–12]. In addition, the clinical and cost-effectiveness of a six-month recall has been questioned by changes in the epidemiology of oral diseases coupled with limited resources [13]. Moreover, the recent paradigm shift in caries management approaches from repair and restoration to prevention and conservation in the form of minimally invasive dentistry also contributes to this debate [13].

Evidence reveal that the recall interval between dental examinations should be tailored to the patient's needs and risk indicators since some patients may need examinations sooner than six months [11, 14, 15]. The risk-based approach for selecting recall interval period has been adopted in several countries; for example, in the United States, the standard recall interval period for low -caries-risk subjects is from 12 to 24 months. In Australia, the school dental service has implemented a recall system based on the dentist's evaluation of an individual caries risk [16, 17]. In England and Wales, the National Institute of Health and Care Excellence (NICE) has prescribed that the recall interval period must be designed separately for every individual according to the assessment of the disease level [18].

Globally different recall intervals are recommended depending on the basis of age group and caries risk intensity. The guideline by NICE recommends that in cases of individuals less than 18 years, the maximum interval is 12 months, and the frequency of recall intervals are 3, 6, 9, and 12 months [18]. Furthermore, the American academy of pediatric dentistry recommends a recall interval of 12–24 months for low risk, 6–12 months for moderate risk, and 3–6 months for children ≥ 6-year-old [19].

Caries risk assessment (CRA) is a vital part of a patient-centered approach to managing caries [20]. Caries risk assessment assists clinicians in determining patient risk for dental caries and determining the appropriate recall interval for treatment. Cariogram, a computerized program that determines caries risk, has proven effective in evaluating caries risk [21]. Cariogram demonstrates the link between caries and its associated factors. It also clarified the chance to avoid caries, produced a graphical presentation of caries risk, and prescribed preventive treatments [21]. Applying a full Cariogram in epidemiological surveys is not feasible as it requires bacterial and saliva testing, which are expensive and cannot be obtained immediately [22]. Reduced Cariogram, which eliminate laboratory tests, are as reliable as conventional Cariogram. Additionally, there is evidence that reduced Cariograms can be used to identify caries risk in preschoolers, school children, communities and in clinical practices [23, 24]. Further evidence shows that reduced Cariograms perform slightly better than full Cariograms. Moreover, it requires fewer resources and time [24].

In Pakistan, no recommended guidelines are available regarding the dental recall interval for children. Dental recall visits in Pakistan are mainly influenced by dentist preferences. These dental visits are usually fixed or based on recalls at six months or problem-oriented visits. A patient who has a problem-oriented appointment usually comes to the dental clinic when they experience unbearable pain or difficulty in chewing. As mentioned previously, fixed or six-monthly dental visits are scientifically inappropriate.

In Pakistan's oral health care services, dental caries is measured using WHO methods (DMFT/DMFS) [25]. These methods are mainly based on treating dental caries through dental fillings rather than prevention. However, evidence shows that treating tooth cavitation through dental fillings fails to prevent and treat rapidly spreading dental caries [25]. The isolated studies in Pakistani 11–12-year-old children revealed a mean DMFT score of 1.2 to 2.5, which is considered low [26–28]. The existing evidence shows a relationship between DMFT scores and caries progression, indicating that when the DMFT score is low, progression rates are slower while they are more rapid in a population with higher DMFT scores [29]. Therefore, slow caries progression, making the traditional six-month recall interval inappropriate for the caries management of this population.

In Pakistan, the low allocation of resources and lack of financial capacity make disease management difficult. Existing oral health services must be reoriented to focus on less expensive technology in order to treat the population that has a genuine need for dental care. Hence, to make the most effective use of limited resources, it is necessary to determine the appropriate recall interval for caries management based on the risk intensity of caries. The aim of this is to determine the rate of caries increment among 11–12-year-old Pakistani children over 18 months using Modified International Caries Detection and Assessment Systems II (ICDAS) and subsequently to establish an appropriate dental recall interval for this targeted population according to their caries risk intensity.

## Materials and methods

### Study design

The prospective longitudinal study was conducted in Bhakkar city of Punjab, Pakistan. Among the 200 schools in Bhakkar, seven schools with the highest student enrolment were conveniently selected. The study design included a caries risk assessment, a baseline caries measurement, and three follow-up visits for subsequent caries measurements. Each follow-up for caries measurement had an interval of six months, up to 18 months. Intraoral examinations were carried out within the school premises. During examinations, children identified with cavitated carious lesions and needing urgent care were referred to the nearest district headquarter hospital (DHQ) for treatment.

Before baseline and follow-up visits, caries risk assessment was performed on seven factors indicated in the computer-based reduced Cariogram program. Saliva and bacterial testing were excluded. A three-day diet diary was used to estimate the frequency and content of meals. Having collected and inserted all data into the Cariogram, the computer program categorised each child into a low, moderate, or high caries risk group.

### Ethical approval

This study protocol was reviewed and approved by The Medical Ethics Committee of the Faculty of Dentistry of Universiti Malaya, Kuala Lumpur, Ref no: DF 71 CO1512/0072(P). The district education officer approved the study in public-sector schools, while separate approvals were obtained from the administrations of private-sector schools. Written informed consent was obtained from the participant's parents. All methods were performed in accordance with the Declaration of Helsinki and relevant guidelines and regulations.

### Participants

Students aged 11 to 12-year-old from each school were contacted and invited to participate. Those included were public- and private-sector students aged 11 to 12 years who understood basic instructions for compliance purposes. Children under or above the age of 11 to 12 years, people with acquired physical disabilities, and those receiving orthodontic treatment were excluded.

### Sample size

The sample size was calculated based on the effect size mentioned in the literature [30] and the formula established by Chinna & Krishnan [31]. The final sample size of 60 participants was selected for each caries risk group (low, moderate and high). The minimum sample size required was 180, and by considering a 20% attrition rate, the sample size needed was at least 226 participants.

### Calibrations

A single examiner underwent training and calibration procedures for the ICDAS. Training for the modified ICDAS index regarding the caries coding procedure was provided by the ICDAS Task Force Committee member based at the Faculty of Dentistry, University Malaya. The training consisted of completing the 90-min ICDAS *e*Learning Program, a 90-min lecture, assessing clinical images and scoring coronal surfaces of posterior extracted teeth representing all ICDAS scores (Codes 0–6). All visual examinations were conducted using optimal clinical facility/equipment during calibration, including dental mirrors, magnified dental loupes, headlights, and ball-ended probes.

After the training exercise, a calibration process was carried out over two days with a time interval of one week. The ICDAS codes for the 62 mounted teeth used in the calibration process have been validated by the aforementioned task group. Teeth were recorded as follows: 0: sound; 1: first visual change in enamel seen after drying; 2: distinct visual change in enamel; 3: localised enamel breakdown with no visible dentin; 4: underlying dark shadow from dentine; 5: distinct cavity with visible dentine; 6: extensive distinct cavity with visible dentin [32]. The intra- and inter-examiner reliability were assessed using Cohen’s weighted kappa. The inter-examiner kappa value achieved was 0.69, and the intra-examiner kappa value was 0.82 and 0.97 (Wt Kappa) for the reference examiner.

### Instrument and clinical examination

The equipment used in this study includes an adjustable portable dental chair, and the instruments used for intraoral examination were mouth mirror, ball-ended periodontal probes, tweezers, and LED headlight illumination mounted on dental loupes of 3.5 magnification and cotton roll/gauze.

In the present study, caries measurements were carried out using the modified ICDAS II index specifically designed for epidemiological research. In modified ICDAS teeth were recorded as 0: sound, code A: for initial carious lesions (in modified ICDAS codes 1 and 2 are merged and assigned code A since compressed air could not be used to dry tooth surfaces at the study site) [33, 34], 3: localised enamel breakdown with no visible dentin; 4: underlying dark shadow from dentine; 5: distinct cavity with visible dentine; 6: extensive distinct cavity with visible dentin [32].

In the present study the ICDAS code 3 lesions were considered cavitated since enamel breakdown or micro cavitation is limited to enamel at this stage, and it is irreversible [35]. Further, radiographic evidence indicates that, at this stage, radiolucency is restricted to the outer third of the dentin [36]. Additionally, these lesions require minimally invasive surgical management with no or minimal dentin removal [36].

A single calibrated examiner conducted all the intra-oral examinations. The intra-oral examination was conducted with the children seated on a portable dental chair. The trained dentist performed inspections using LED headlight illumination mounted on dental loupes of 3.5 magnifications. Tooth assessment was initiated from the back of the upper right quadrant. First, the mesial tooth surface was examined, followed by occlusal, distal, buccal, and lingual surfaces. Cotton rolls were used to dry tooth surfaces to examine non-cavitated lesions and a ball-ended periodontal probe to check for surface contours and minor cavitation.

### Dental caries increment estimation

Caries increment among cavitated lesions was measured by modified Beck's method or adjusted caries increment (ADJCI) [37]. The observation unit used was the tooth surface. Three transitions were used to measure caries increment progression, regression, and no progression and no regression.

The transitions used for caries increment are given below,

1. Progression

- Progression from a sound surface to non-cavitated decay.
- Progression from the sound or noncavitated decay to missing and crowned.
- Progression from noncavitated decay associated with filling to cavitated decay and cavitated decay associated with filling.

2) Regression

- Regression from non-cavitated decay or cavitated decay to sound surface.
- Regression from non-cavitated decay associated with filling to sound.
- Regression from cavitated decay associated with filling to sound or non-cavitated decay associated with filling.
- Regression from filled to sound.

3) No progression and no regression

- No change in caries status between baseline and follow-up caries assessment
- Transition from missing tooth surface regardless of the subsequent assessment of caries.
- Transition from cavitated decay or cavitated decay associated with filling.

The count of surfaces with progression, regression, and count of surfaces with no change was inserted into the formula instead of scoring system to calculate ADJCI value at the child level as recommended by Ismail et al., (2011).

$${\text{ADJCI}} = \frac{{{\text{Progression }} \times {\text{ no progression nor regression}}}}{{{\text{Regression }} + {\text{ no progression nor regression}}}}$$

### Statistical analysis

Data were analysed using SPSS 17. Descriptive analysis was performed to estimate the number of surfaces with modified ICDAS scores on the baseline and on follow-ups. A Chi-square test was performed to assess the frequency of participants according to sociodemographic characteristics and caries risk category. Two ICDAS II cutoffs were created for the analysis of cavitated lesions (ICDAS code 3–6) and cavitated plus non-cavitated lesions (ICDAS code A-6). Repeated measure analysis of variance (ANOVA) with a post hoc test using Bonferroni correction was used to compare mean caries increment occurred at 6 months with the caries increment occurred at 12, and 18 months of follow-up. Multiple imputations were performed using Stata 14 to generate plausible data for missing participants to estimate the caries increment. The level of significance was set at less than 0.05.

## Results

In all, 400 children were invited to participate in the study based on the list of names. Of these, 300 children obtained parental consent, 70 refused to participate, and four left school. The final sample size was 226 children, with an average of 57 students per school. The number of attendees, absentees, and dropouts in each visit are shown in Table 1. The proportion of missing data was 36.2%, due to which the power of the sample size was reduced to 70%.

**Table 1:** Follow-up rate and dropout rate at risk assessment, baseline and follow-up examinations

Examination	Number of enrolled participants	Total	Low (*n*)	Medium (*n*)	High (*n*)
Risk assessment	226	226	90	69	67
Baseline caries assessment	Attendees	220	86	67	65
	Absentees	6	2	2	2
	Total Response rate	218/226 = 97%			
1st Follow up	Attendees	174	75	49	50
	Absentees	22	7	8	7
	Dropouts	30	8	12	10
	Total Response rate	174/226 = 77%			
2nd Follow up	Attendees	174	74	49	51
	Absentees	11	3	5	3
	Dropouts	41	13	15	13
	Total Response rate	174/226 = 77%			
3rd Follow up	Attendees	183	75	54	54
	Absentees	0	0	0	0
	Dropouts	43	15	15	13
	Total Response rate	185/226 = 81%			

The distribution of tooth surfaces with modified ICDAS scores on the baseline and on follow-ups are shown in Table 2. The comparison of sociodemographic variables against caries risk groups, as categorised by the Cariogram, is shown in Table 3. At the risk assessment stage, 39.8% of the children were classified as low risk, 30.5% as medium risk, and 29.7% as high risk. Among low-risk participants, a significantly higher number of children were from private schools (76.6%) compared to government-funded schools (20.8%) (*p* = 0.0001).

**Table 2:** Distribution of tooth surfaces with modified ICDAS scores on baseline and on follow ups

Modified ICDAS code	Sound *n* (%)	A *n* (%)	3 *n* (%)	4 *n* (%)	5 *n* (%)	6 *n* (%)	Total *n* (%)
Baseline	27,716 (97.5)	515 (1.81)	89 (0.3)	26 (0.09)	55 (0.19)	14 (0.04)	28,415 (100)
1st Follow up	21,473 (96.8)	464 (2.09)	143 (0.64)	17 (0.07)	52 (0.23)	11 (0.04)	22,160 (100)
2nd Follow up	21,753 (97.1)	414 (1.84)	133 (0.59)	22 (0.09)	66 (0.29)	11 (0.04)	22,399 (100)
3rd Follow up	22,708 (96.8)	379 (1.61)	197 (0.84)	59 (0.25)	60 (0.25)	32 (0.13)	23,435 (100)

**Table 3:** Sociodemographic characteristic of the study sample at baseline (*N* = 226)

Sociodemographic background	Caries risk levels according to cariogram			*p*-value
	Low *n* (%)	Medium *n* (%)	High *n* (%)	
All subjects	90 (39.8)	69 (30.5)	67 (29.7)	
*Gender*				
Male Female	49 (39.8) 41 (39.8)	38 (30.9) 31 (30.1)	36 (29.3) 31 (30.1)	0.98
*Residence*				
Urban Rural	77 (41.4) 13 (32.5)	53 (28.5) 16 (40)	56 (30.1) 11 (27.5)	0.34
*School type*				
Public funded Private funded	31 (20.8) 59 (76.6)	63 (42.3) 6 (7.8)	55 (36.9) 12 (15.6)	0.0001

Chi-square test

The caries prevalence of cavitated and non-cavitated lesions was 74% at baseline, 84% at first follow-up, 81% at second follow-up, and 83% at third follow-up. After 18 months, 73% of the high, 59% of the medium, and 41% of the low-risk children show caries increment.

Caries increment at both cutoff points increased with caries risk at all follow-ups. The highest caries increment was recorded at the third follow-up among high-risk children at cutoff 3–6 (1.95 ± 3.18) and A-6 (4.01 ± 4.31). However, the lowest caries increment was recorded at the third follow-up among low-risk children at cutoff 3–6 (0.18 ± 1.42) and A-6 (1.11 ± 3.33). Total caries increment increases at a cutoff point 3–6 was (0.49 ± 1.45) at the first follow-up, (0.66 ± 2.35) at the second follow–up, and (0.86 ± 2.39) at the third follow-up, respectively. At cutoff A-6, caries increment was (1.99 ± 3.05) at first follow up, (2.24 ± 3.60) at second follow–up, and (2.30 ± 4.05) at third follow-up, respectively.

The comparison of mean caries increments that occurred at all follow-ups within each risk level compared to baseline is shown in Table 4. Analysis reveals that at cutoff 3–6, in a high-risk category, mean caries increment was higher statistically at all follow-ups than baseline. At cutoff A-6 within all risk categories, mean caries increment was statistically higher at all follow-ups as compared to baseline.

**Table 4:** Comparing mean caries increment that occurred 6 months with the caries increment occurred at 12 and 18 months within each risk category at all follow-ups

Risk Category	Caries increment (6 months) Mean ± SD	Caries increment (12 months) Mean ± SD	*p*-value	Caries increment (18 months) Mean ± SD	*p*-value
*Cavitated lesion (3–6)*					
Low risk	0.02 ± 0.60	0.00 ± 0.79	1.00	0.18 ± 1.42	1.00
Medium risk	0.40 ± 1.35	0.45 ± 1.82	0.24	0.70 ± 2.17	0.05
High risk	1.22 ± 2.01	1.78 ± 3.57	0.01*	1.95 ± 3.18	0.01*
*Non-cavitated plus cavitated lesion (A-6)*					
Low risk	0.84 ± 1.92	1.21 ± 3.38	0.006*	1.11 ± 3.33	0.01*
Medium risk	2.49 ± 3.29	2.41 ± 3.30	0.84	2.48 ± 4.11	0.98
High risk	3.01 ± 3.54	3.47 ± 3.82	0.01*	4.01 ± 4.31	0.01*

Repeated measures ANOVA post hoc analysis

Statistically significant at *p* < 0.05*

## Discussion

Longitudinal studies conducted on school children can provide useful estimates of dental caries increment over a period of time [38]. To the extent of our knowledge, longitudinal studies assessing caries increment among children with different caries risk categories have not been investigated on the Pakistani population; thus, comparison with the current research at the national level is impossible. In contrast, studies on non-Pakistani school-age population report caries increment only among cavitated lesions [23, 39, 40]. In our study, caries increment was measured at a cutoff 3–6 and cutoff A-6 for each caries risk category (Table 4). So far, only Ismail et al.'s 2011 study has estimated caries increment on both cavitated and non-cavitated lesions [37]. However, this study did not show a caries risk assessment since they only evaluated the primary dentition. Therefore, our findings cannot be compared with the findings of that study.

In this study, the recommendation of recall intervals for each risk level was made by comparing the mean caries increment that occurred at all follow-ups within risk categories at cutoff 3–6 (only cavitated lesions were considered) and cutoff A-6 (both cavitated and non-cavitated lesions were considered). The dental caries increment was used at both cutoff points because the caries progression from enamel to dentine plays a fundamental role in planning recalls [29].

Among participants with low and medium caries risk, at cutoff 3–6, no significant differences were observed in mean caries increment, indicating that progression in cavitated lesion did not occur until 18 months (Table 4). Hence, for managing cavitated carious lesions in low and medium-risk children, recall intervals can be extended up to at least 18 months. A recall interval of fewer than 18 months has been recommended for low and medium-caries risk children in Sao Paulo and Scotland [39, 41]. Shortening the interval between dental recalls for children with a low risk of caries may not provide significant benefits in terms of their overall experience with the disease [42]. In contrast, they may face financial repercussions, such as lost appointment time and the increased likelihood of iatrogenic interventions [42].

Based on the caries increment in children with high caries risk at cutoff 3–6, the difference in mean caries increment was statistically significant from the six-months follow-up onwards, indicating early caries progression (Table 4). As a result, recall intervals should not be extended beyond six months in high-risk children to manage cavitated carious lesions.

At cutoff A-6, where both non-cavitated and cavitated lesions were considered, mean caries increment was statistically significant in low and high risk categories at all follow-ups (Table 4). However, in a medium risk category no statistical significant difference was found at all follow-ups. The possible explanation of reduced caries increment in medium risk participants is the regression of early carious lesion. Furthermore the evidence shows that average risk individuals show little or no caries development for longer duration and unexpected occurrence of the carious lesion [48]. Based on caries increment at cutoff A-6, the recall interval should not be extended beyond six months to manage cavitated and non-cavitated carious lesions in low, moderate and high-risk children.

In the present study, the follow-ups were made at six months from baseline. However, for children in the higher-risk categories, there is a possibility that caries may have occurred even earlier than six months from baseline. If this is the case, caries recall intervals should be made earlier than six months from baseline [15, 35]. Similarly, the public oral health system in Sao Paulo recommends a recall interval of 4 months between dental examinations [39].

In Pakistan, where only cavitated lesions are measured to estimate caries, the recall interval can be extended up to 18 months for children with low and medium caries risk. Furthermore, for children with a high caries risk, the risk-based recall interval cannot be extended beyond six months because new caries lesions may go undetected and thus untreated. On the other hand, if ICDAS II is adopted in Pakistan for caries detection in the future, the recall interval of six months for low, moderate and high risk children regardless of caries risk, can be adopted for caries management among 11–12-year-old Pakistani children. However, the adoption of ICDAS II with a 6 month recall interval in the future cannot negate the use of risk assessment tools because diagnostic and treatment approaches vary based on the risk category.

The limitations of this study include the lack of radiographic evaluation for the detection of interproximal carious lesions. Therefore, the prevalence of caries and subsequently the calculated caries increment rate may not truly reflect the caries experience of the study population. Moreover, the caries increment rate may be underestimated because ADJCI cannot differentiate between biologically plausible and biologically implausible reversals. This study was conducted in a specific geographical area, and convenience sampling was used. As a result, the findings may not be generalisable to the entire population. However, the results can be applied to a population with similar characteristics.

Caries estimation was performed at the child level, accurately reflecting disease initiation in subjects. In contrast, previous studies report caries increment only for cavitated lesions [43–47]. However, the inclusion of non cavitated lesions used in this study to achieve an accurate caries progression rate is the strength of our research.

## Conclusion

Based on evidence from the current study, rapid caries progression rates were observed among individuals with higher caries risk. The recommended risk-based recall interval for caries management in cavitated lesions of Pakistani 11–12-year-old children has been suggested at 18 months for those with low and medium caries risk and at six months for those classified in the high caries risk group. Recommendations for risk-based recall intervals for caries management in non-cavitated and cavitated lesions are six months for low-risk, moderate risk and high-risk Pakistani 11–12-year-old children.

## Data Availability

The datasets used and analysed during the current study are available from the corresponding author upon reasonable request.

## Abbreviations

ICDAS: International caries detection and assessment systems
ADJCI: Adjusted caries increment
